# Comparative Transcriptome Analysis Identifies Candidate Genes Related to Black-Spotted Pattern Formation in Spotted Scat (*Scatophagus argus*)

**DOI:** 10.3390/ani11030765

**Published:** 2021-03-10

**Authors:** Xiaozhan Lin, Changxu Tian, Yang Huang, Hongjuan Shi, Guangli Li

**Affiliations:** 1 Fisheries College, Guangdong Ocean University, Zhanjiang 524088, China; linxiaozhan@stu.gdou.edu.cn (X.L.); tiancx@gdou.edu.cn (C.T.); zjouhy@126.com (Y.H.); shihongjuan1990@163.com (H.S.); 2 Guangdong Research Center on Reproductive Control and Breeding Technology of Indigenous Valuable Fish Species, Guangdong Provincial Engineering Laboratory for Mariculture Organism Breeding, Guangdong Provincial Key Laboratory of Pathogenic Biology and Epidemiology for Aquatic Economic Animals, Guangdong Ocean University, Zhanjiang 524088, China; 3 Southern Marine Science and Engineering Guangdong Laboratory (Zhanjiang), Zhanjiang 524088, China

**Keywords:** Illumina sequencing, pigment synthesis, molecular mechanisms, skin coloration

## Abstract

**Simple Summary:**

Spotted scat (*Scatophagus argus*) is a commercially important marine aquaculture and ornamental fish species in China and East Asian countries. There are dozens of black spots on each side of the body, and the caudal fin, which is yellow and black, is appreciated in ornamental fish markets. To explore the genetic mechanisms of its pattern formation, we found 2357 differentially expressed genes (DEGs) by comparing the transcriptome in the black-spotted skin, non-spotted skin and caudal fin in *S. argus*. The results will expand our knowledge about the molecular mechanism of important genes and pathways associated with pigment pattern formation and provide a certain theoretical basis for the molecular breeding in *S. argus*.

**Abstract:**

Spotted scat (*Scatophagus argus*) is an economically important marine aquaculture and ornamental fish species in Asia, especially in southeast China. In this study, skin transcriptomes of *S. argus* were obtained for three types of skin, including black-spotted skin (A), non-spotted skin (B) and caudal fin (C). A total of nine complementary DNA (cDNA) libraries were obtained by Illumina sequencing. Bioinformatics analysis revealed that 1358, 2086 and 487 genes were differentially expressed between A and B, A and C, and B and C, respectively. The results revealed that there were 134 common significantly differentially expressed genes (DEGs) and several key genes related to pigment synthesis and pigmentation, including *tyrp1*, *mitf*, *pmel*, *slc7a2*, *tjp1*, *hsp70* and *mart-1*. Of these, some DEGs were associated with pigmentation-related Kyoto Encyclopedia of Genes and Genomes (KEGG) pathways, such as tyrosine metabolism, melanogenesis, the Wnt signaling pathway and the mitogen-activated protein kinase (MAPK) signaling pathway. The results will facilitate understanding the molecular mechanisms of skin pigmentation differentiation in *S. argus* and provide valuable information for skin coloration, especially the formation of spotted patterns on other marine fish species.

## 1. Introduction

Skin pigmentation and color patterns are the most diverse phenotypic characteristics in animals. Their functions are closely related to adaptation to different environments, including temperature regulation, camouflage, social interaction, spouse choice, etc. [1,2,3]. The formation of color patterns is determined by various pigments synthesized by chromophores or pigment cells. The color of pigment cells and pigment pattern formation and maintenance are related to the nutritional, physiological, genetic and environmental factors, making pigmentation a complex biological process [4]. Of these, the genetic basis is the most important factor affecting skin pigmentation through gene regulation. At present, genetic and genomic analysis techniques are used to explore the mechanisms of skin pigmentation.

Six types of pigment cells have been reported in vertebrates. Among them, only melanocytes are found in the skin of mammals, and only teleost fishes have six types of pigment cells, including melanophores (black, dark brown), xanthophores (yellow), erythrophores (red/orange), iridophores (reflecting), leucophores (white) and cyanophores (blue) [5,6,7,8]. These chromatophores synthesize and retain their specific pigments and inner structures intracellularly [9]. Skin pigmentation and pattern reflect the numbers and arrangements of the chromatophores. Melanophores and other types of pigment cells are also focused on pattern formation. Melanophores and melanin are the main factors that determine skin color. The molecular melanin biosynthesis mechanism has been widely studied in vertebrates. The melanogenesis pathway is conservative [10,11]. Melanin synthesis takes place within the melanosomes of melanocytes and involves the tyrosine metabolism pathway. Tyrosinases (TYRs), tyrosinase-related protein 1 (TYRP1) and dopachrome tautomerase (DCT) are important enzymes in melanin synthesis [12,13]. The increased expression of the microphthalmia-associated transcription factor (MITF) and its activation by phosphorylation stimulate the transcription of TYR, TYRP1 and DCT [12,14]. However, the biosynthesis of other types of pigments via different pathways and regulatory networks is less studied [6]. Several reports were recorded about pigmentation on model organisms such as medaka (*Oryzias latipes*) and zebrafish (*Danio rerio*) [15,16].

Spotted scat (*Scatophagus argus*) is a kind of edible and ornamental fish. It is a commercially important marine species in China and East Asian countries owing to its high nutrition and delicate flavor. There are dozens of black spots on both sides of the fish body, and the caudal fin is yellow and black. The molecular mechanisms underlying the formation of the body color, especially the black spots in *S. argus,* are not well understood. We used RNA-Seq to detect differentially expressed genes (DEGs) by comparing the black-spotted skin, non-spotted skin and caudal fin in *S. argus*. The study aimed to detect the important genes and pathways associated with black-spotted pigment pattern formation in *S. argus*.

## 2. Materials and Methods

### 2.1. Ethical Statements

This study was approved by the Institutional Animal Care and Use Committee (IACUS) of Guangdong Ocean University (Zhanjiang, China). Sampling procedures complied with the guidelines of the IACUS on the care and use of animals for scientific purposes.

### 2.2. Sample Preparation

**Figure 1:**
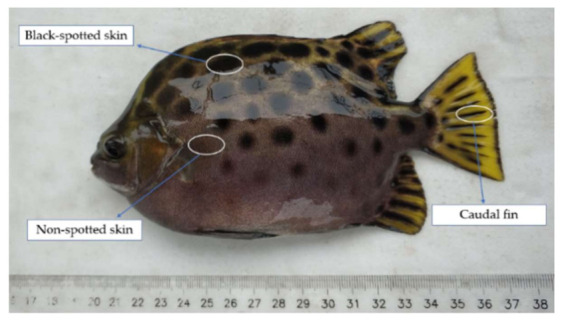
Sampling distribution of different color parts of *S. argus* (black-spotted skin; nonspotted skin; caudal fin).

The experimental fishes (*S. argus*) (Figure 1, total length: 17.27 ± 1.52 cm; body weight: 147.90 ± 14.62 g) were obtained from Donghai Island, Guangdong, China, and were kept in an outdoor cement pool with water (water temperature: 25 °C) for one week. Black-spotted skin (A), non-spotted skin (B) and caudal fin (C, contained both black and yellow areas) tissues (0.8–1 cm^2^, 40–50 mg) were collected and stored in liquid nitrogen for RNA isolation. The same tissues of every three fishes were used as a sample group, forming a total of nine libraries.

### 2.3. RNA Extraction and Illumina Library Preparation

The total RNA (n = 3 per group) from the three groups was extracted using TRIzol reagent (Life Technologies, Carlsbad, CA, USA) following the manufacturer’s instructions. The RNA purity (Table S1) was monitored by Nano spectrophotometer (Nanodrop 2000c, Thermo Scientific, Wilmington, DE, USA). The RNA integrity was checked by ethidium bromide staining of 28S and 18S ribosomal bands on a 1.0% agarose gel. Then, a total of 3 μg of RNA was prepared for each Illumina library sequence. Sequencing libraries were generated using the NEBNext^®^ Ultra™ RNA Library Prep Kit for Illumina^®^ (NEB, New England Biolabs, Palo Alto, CA, USA) following the manufacturer’s instructions. The library fragments were purified using the AMPure XP system (Beckman Coulter, Beverly, MA, USA) to select 250–300 bp complementary DNA (cDNA) fragments, and each library quality was assessed by the Agilent Bioanalyzer 2100 system (Agilent Technologies, Palo Alto, CA, USA). Then, 3 µL of USER Enzyme (NEB, New England Biolabs, Palo Alto, CA, USA) was used with size-selected, adaptor-ligated cDNA at 37 °C for 15 min, followed by 5 min at 95 °C before PCR. PCR was performed with Phusion High-Fidelity DNA polymerase, universal PCR primers and Index (X) Primer. Finally, PCR products were purified by the AMPure XP system and the library quality was assessed by the Agilent Bioanalyzer 2100 system. All clean libraries of sequencing data were submitted to the NCBI Sequence Read Archive (SRA) database (Accession No.: PRJNA670377).

### 2.4. Data Filtering, Reads Mapping and Differential Expression Analysis

Based on the *S. argus* reference genome and gene model annotation files (unpublished data), an index of the reference genome was built, and the paired-end clean reads were aligned to the reference genome using Hisat2 v2.0.5 (https://anaconda.org/biobuilds/hisat2) (accessed on 16 August 2020). The gene expression levels were estimated by fragments per kilobase of exon model per million reads mapped (FPKM) [17]. Clean reads were picked out by removing reads containing adapter, poly-N and low-quality reads from raw data, which were processed through in-house perl scripts. DESeq2 R package (version 1.16.1) was used to identify DEGs [18] with a threshold of |log_2_ fold change|≥2.0 and p-value (padj) <0.05 [19]. The DEGs were mapped to the Kyoto Encyclopedia of Genes and Genomes (KEGG) (http://www.genome.jp/kegg/)) (accessed on 22 September 2020) and Gene Ontology (GO) databases (padj ≤ 0.05).

### 2.5. Validation of DEGs by Quantitative Real-Time Polymerase Chain Reaction (qRT-PCR)

A total of 14 DEGs were randomly selected from black-spotted skin vs. non-spotted skin, black-spotted skin vs. caudal fin, and non-spotted skin vs. caudal fin to verify the expression of DEGs. The primers of the selected genes were designed using Primer Premier software v5.0, which are listed in Table S2. qRT-PCR was performed using SYBR Green qPCR Mix (Dongsheng Biotech, Guangzhou, China) on a Light Cycler real-time quantitative PCR system (Roche, Basel, Switzerland) according to the manufacturer’s instructions. *β-actin* gene was used as a reference in order to standardize the gene expression values. PCR amplification was performed in triplicate for each gene. Relative expression levels were measured in terms of the threshold cycle value and were normalized using the 2 ^−ΔΔCt^ method [20].

## 3. Results

### 3.1. Raw Sequencing Reads and Quality Statistics

Nine cDNA libraries were built from three different body parts of the fish. In Table 1, we determined the raw reads, clean reads, clean bases, Q30 percentages and GC percentages in order to describe the quality of the transcriptomic data from *S. argus*. A total of 485.6 million raw reads were generated after removing the reads, containing adapter, ploy-N reads and the lower quality reads from raw data. We got 478.1 million clean reads, and the percentages of Q30 bases were more than 92% for all the samples, indicating a high-quality sequence.

### 3.2. Comparative Analysis of DEGs

**Figure 2:**
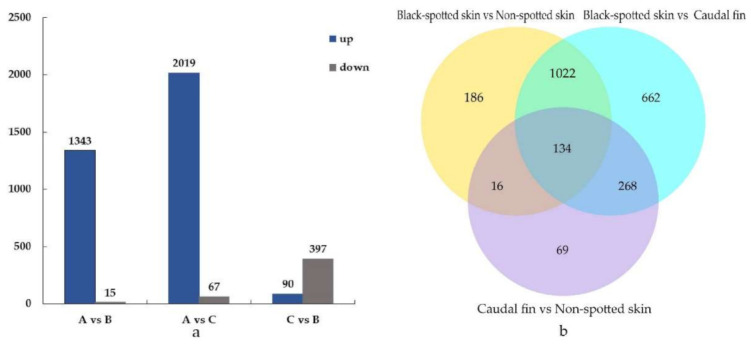
(**a**) Comparison of the number of differentially expressed genes (DEGs) in three different tissues of *S. argus*. The horizontal axis represents the samples on the control of the situation, and the vertical axis measures the difference of gene numbers. There are two expression levels. The blue color stands for the upregulated expression, and the gray color represents the downregulated expression. (**b**) DEG number and Venn diagram of the overlap of the different groups. A (black-spotted skin), B (non-spotted skin) and C (caudal fin).

The results showed that compared with the black-spotted skin, 1358 genes were differentially expressed in the non-spotted skin. Of these, 1343 genes were upregulated and 15 genes were downregulated. In the caudal fin, a total of 2086 genes were differentially expressed, with 2019 genes being upregulated and 67 genes being downregulated. Compared with the caudal fin, 487 genes were differentially expressed in the non-spotted skin. Of these, 90 genes were upregulated and 397 genes were downregulated (Figure 2a). In addition, a total of 134 coexpressed genes showed significant differential expression levels in three different groups (Figure 2b), which may be related to skin coloration and skin derivatives production in *S. argus*.

### 3.3. GO and KEGG Pathway Enrichment Analysis

**Figure 3:**
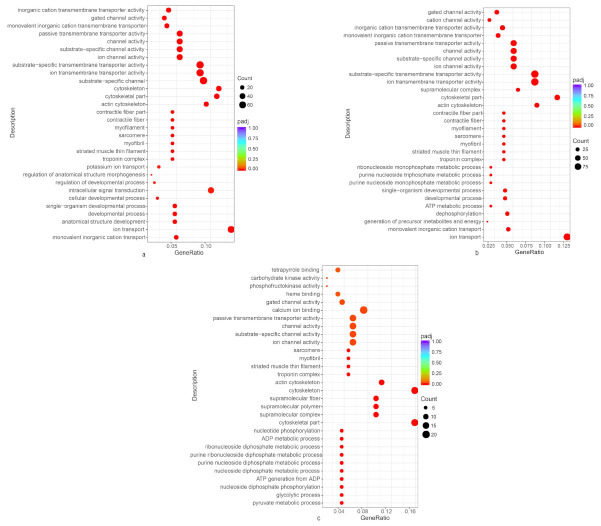
Gene Ontology (GO) function classification of the differentially expressed genes (DEGs) comparison between the groups (padj < 0.05). (**a**) Black-spotted skin vs. non-spotted skin; (**b**) Black-spotted skin vs. Caudal fin; (**c**) Caudal fin vs. non-spotted skin. The x-axis represents the number of genes, and the y-axis represents GO terms.

We made a comparison among the A (black-spotted skin), B (non-spotted skin) and C (caudal fin) transcriptomes to explore whether the mechanism underlying the black-spotted skin color formation of *S. argus* was controlled by multiple agents via pathways. DEGs identified in three different comparisons were used for the GO and KEGG functional enrichment analysis. According to the GO functional analysis, DEGs were classified into three major functional categories, including biological process (BP), cellular component (CC) and molecular function (MF). In each comparison, the top ten most significant enrichment pathways were selected in three different categories. The detailed annotations for each category are depicted in Figure 3a–c. We found that monovalent inorganic cation transport (GO:0015672), ion transport (GO:0006811), pyruvate metabolic process (GO:0006090) and glycolytic process (GO:0006096) were the most enriched components in the BP category; troponin complex (GO:0005861), striated muscle thin filament (GO:0005865), cytoskeletal part (GO:0044430) and supramolecular complex (GO:0099080) were the enriched terms in the CC category; ion transmembrane transporter activity (GO:0015075) and ion channel activity (GO:0005216) were the most common terms in the MF category.

A total of 297 KEGG pathways were found in the study. The top 10 pathways in each comparison were selected, as shown in Table S3. The pathways with most of the represented genes were the oxidative phosphorylation, cardiac muscle contraction, focal adhesion, neuroactive ligand-receptor interaction and calcium signaling pathways. As shown in Table 2, some DEGs were related to pigmentation. We mainly focused on genes related to melanin synthesis, deposition and migration. The KEGG pathway analysis results showed that some DEGs were associated with pigmentation-related pathways, such as tyrosine metabolism, melanogenesis, the Wnt signaling pathway and the MAPK signaling pathway.

### 3.4. Validation of Gene Expression Levels

**Figure 4:**
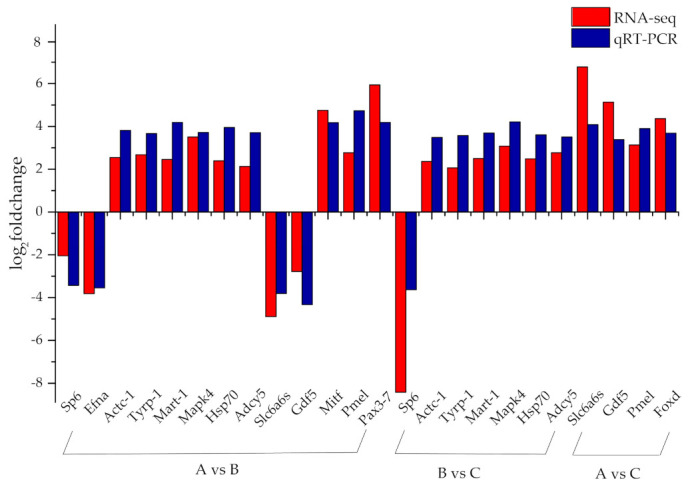
Comparison of gene expression data between RNA-seq and quantitative real-time polymerase chain reaction (PCR) (qRT-PCR). The x-axis presents the gene name, and the y-axis presents a fold change in gene expression.

A total of 14 DEGs were selected and analyzed using qRT-PCR (Figure 4). The changing trends of those genes from qRT-PCR were similar to the results from the RNA-seq expression analysis, which supports the reliability of the transcriptome data.

## 4. Discussion

Different mechanisms are responsible for the changes in skin pigment patterns in animals. Black spot pigment pattern is the most important phenotypic feature of *S. argus.* However, the mechanism of formation is still unknown. We conducted a comparative transcriptome analysis of three differently pigmented regions of the adult black-spotted skin, non-spotted skin and caudal fin to screen for genes potentially involved in the maintenance of a specific pigment pattern in *S. argus*. The results showed that more differentially expressed genes were upregulated in black-spotted skin when compared to non-spotted skin and caudal fin, indicating that the formation process of black spots on the skin was complex and that more genes needed to participate in the process. As shown in Figure 2, there were DEGs and 134 shared coexpressed genes in three comparisons. In addition, the top 30 functional GO terms and top 10 KEGG pathways were enriched in three groups (Figure 3 and Table S3). The KEGG pathway analysis showed that a substantial number of DEGs between the three different regions were associated with pigmentation-related pathways (e.g., MAPK signaling pathway, Wnt signaling pathway, tyrosine metabolism) and cell–cell communication, such as focal adhesion, the calcium signaling pathway and ECM–receptor interaction.

In this study, differential expressions were observed by comparing differently colored regions in *S. argus*, most probably associated with specific expression profiles of individual pigment cell types in a particularly colored skin region [21]. The two most common types of melanin are eumelanin and pheomelanin in melanophore. Eumelanin corresponds to a brown/black color, while pheomelanin corresponds to a red/yellow color. The expression levels of eumelanin-related genes (such as *tyrp1*, *mart-1*, *hsp70*) in black-spotted skin were significantly higher than those in non-spotted skin. After investigating the DEGs from two skin tissues, the expression levels of tyrosinase-related protein 1 (*tyrp1*), melanoma antigen recognized by T-cells 1 (*mart-1*) and tight junction protein 1 (*tjp1*) were upregulated in black-spotted skin, implying that these genes play a key role in the black coloration in *S. argus* in the eumelanin synthesis-related pathway. The expression level of *tyrp1* was upregulated in black-spotted skin compared with non-spotted skin, suggesting that *tyrp1* is a key gene that contributes to the brownish-black pigment pattern in *S. argus* by playing an important role in the eumelanin synthesis pathway. *tyrp1* is expressed specifically in melanocytes, and it plays a crucial role in the formation of pigment patterning by affecting melanin synthesis, stabilizing tyrosinase protein, modulating tyrosinase catalytic activity, maintaining the melanosome structure and affecting melanocyte proliferation and cell death [22,23,24]. Mutations of *tyrp1* cannot form a black coat color in mice [25], dogs [26], cats [27], cows [28] and sheep [29]. *tyrp1* plays similar roles in teleost fish species, and the knockout of *tyrp1* gene in zebrafish and medaka hinder the formation of eumelanin [15]. Our results confirmed the role of *tyrp1* gene in the skin coloration of *S. argus*. It has been shown that *sox10* gene can activate the *mitf* and raise the expression of *mitf* [30]. In mice, both *mitf* and *sox10* are necessary for the expression of *tyr*, but in zebrafish *mitf* is enough for the expression of *tyr* [31]. In Table 2, three forms of adenylate cyclase genes (*adcy5*, *adcy6* and *adcy9*) were significantly expressed when comparing black-spotted skin to non-spotted skin. Adenylate cyclase activator was used to treat primary cultures of normal human melanocytes, and the activation of adenylate cyclase caused *mitf* to start transcription and contributed to pigmentation [32,33]. Our results indicated that the adenylate cyclase genes may play an important role in the process of promoting melanin synthesis for fish. *tjp1*, encoding a tight junction protein, was upregulated in black-spotted skin when compared with non-spotted skin, suggesting that the expression of this gene could affect the formation of pigment patterning. In a previous study, a zebrafish with a mutation functional *tjp1a* protein was examined [34]. It was observed that *tjp1a* was upregulated, as expressed in dense iridophores, but downregulated in loose-form iridophores. The mutant had a spotted pattern rather than the usual striped pattern because the dense iridophores interrupted the dark stripes, resulting in a spotted pattern. Another role of *tjp1* gene in skin pigment patterning is that tjp1 protein is a scaffolding protein that links tight junction proteins to the actin cytoskeleton, and could affect the rearrangement of the cytoskeleton and the movement of the cells [35]. There were several longitudinal black stripes on both sides of the body before the formation of black spots, which may be related to the expression of *tjp1* gene. We hypothesized that *tjp1* might play a distinct role insofar as iridophores fix a lot of melanophores in a specific position and form black spots. However, further studies are needed.

We found that the expression levels of premelanosome protein (*pmel/silv*), solute carrier family 7 member 2 (*slc7a2*) and microphthalmia-associated transcription factor (*mitf*) were higher in black-spotted skin. *pmel* is a significant eumelanin synthesis pathway gene, encoding the premelanosome protein, which is extensively expressed in pigment cells [36]. Mutations in *pmel* gene lead to pigmentation phenotypes in many vertebrate species. In mice, *pmel* is required to regulate the normal development of skin and choroidal melanocytes and retinal pigment epithelial cells [36]. In chicken, the mutation of *pmel* inhibited the synthesis of eumelanin in the skin and plumage [37]. Similar results were obtained in horses, in which *pmel* mutations reduced the concentration of black pigment in the mane and tail [38]. Our results showed that the expression level of *pmel* in black-spotted skin was higher than that in the caudal fin. The results further confirmed the role of *pmel* in the eumelanogenesis of *S. argus*.

In addition, compared with non-spotted skin and caudal fin, DEGs identified in black-spotted skin were significantly enriched in actin cytoskeleton and cytoskeletal proteins. Cytoskeleton proteins are essential in mechanical transduction, as the cytoskeletal network promotes the translocation of signal molecules from focal adhesion sites to the cytoplasm [39]. The pigmentation-related pathways showed significant enrichments in the black-spotted skin. We speculate that the pigmentation of black spots in fish occurred because cytoskeleton construction induced by the skeletal muscle cell movement could cause the contraction and gathering of pigment cells and promote the coloration of the skin, which explains the change in pattern from stripes to spots from the juvenile to the adult stage of the fish. We noticed that there was a stage where the caudal fin only had a yellow pattern, and we speculated that black spots gradually migrated from the skin to the caudal fin and that the black spot was separated into the current black pattern. However, further investigation is still needed to confirm our conjecture.

## 5. Conclusions

We sequenced transcripts from the skin and caudal fin of spotted scat for the first time and identified 2357 DEGs (with a fold change ≥2). These DEG data provide a reference for screening genes for their effect on pigment patterning in *S. argus*. The results significantly enhanced our understanding of the composition of the *S. argus* skin transcriptome and the potential differences in gene expression associated with skin pigment pattern maintenance, especially the formation of black spots. Our results suggested an important role of junctions and cytoskeleton rearrangements in maintaining *S. argus* skin pigment patterns due to differential gene expression, providing a certain theoretical basis for molecular breeding in the future.

## Figures and Tables

**Table 1:** Summary of the transcriptomic data from *S. argus*.

Group	Raw Reads	Clean Reads	Clean Bases (G)	Q30 (%)	GC Content (%)
Black-spotted skin (A)					
A1	58,647,296	57,844,474	8.68	93.16	46.97
A2	54,843,424	53,929,484	8.09	92.84	46.67
A3	61,189,662	60,096,498	9.01	94.12	46.56
Non-spotted skin (B)					
B1	55,148,934	54,250,656	8.14	92.72	45.94
B2	53,000,358	52,199,276	7.83	92.69	46.70
B3	56,077,298	55,093,088	8.26	92.64	46.07
Caudal fin (C)					
C1	46,402,098	45,867,418	6.88	92.16	46.15
C2	50,405,758	49,913,528	7.49	92.47	46.31
C3	49,958,956	48,909,024	7.34	94.57	46.88

**Table 2:** Analysis of differentially expressed genes (DEGs) related to pigmentation.

Gene	Gene ID	Note	Log_2_ Fold Change
Black-spotted skin vs. Non-spotted skin			
*Tyrp1*	EVM0000142	Tyrosinase-related protein 1	2.68
*Wnt2*	EVM0010869	Wingless-type MMTV integration site family, member 2	8.04
*Camk2*	EVM0018979	Calcium/calmodulin-dependent protein kinase	2.75
*Adcy5*	EVM0008013	Adenylate cyclase 5	2.12
*Adcy6*	EVM0004537	Adenylate cyclase 6	2.76
*Wnt11*	EVM0010741	Wingless-type MMTV integration site family, member 11	5.96
*Foxd*	EVM0009699	Forkhead box protein D	4.67
*Mapk4*	EVM0001218	Mitogen-activated protein kinase 4	3.51
*Adcy9*	EVM0003371	Adenylate cyclase 9	2.29
*Hgf*	EVM0021385	Hepatocyte growth factor	2.29
*Tgfb3*	EVM0004513	Transforming growth factor beta-3	2.96
*Rasgrf1*	EVM0005858	Ras-specific guanine nucleotide-releasing factor 1	4.77
*Efna*	EVM0012046	Ephrin-A	−3.81
*Mart-1*	EVM0011519	Melanoma antigen recognized by T-cells 1	2.45
*Tjp1*	EVM0021943	Tight junction protein 1	2.12
*Hsp70*	EVM0013804	Heat shock 70 kDa protein 12A isoform X1	2.39
Black-spotted skin vs. Caudal fin			
*Pmel*	EVM0007408	Premelanosome protein	2.23
*Slc7a2*	EVM0021987	Solute carrier family 7 member 2	4.29
*Pax3*	EVM0000393	Paired box protein 3	2.45
*Pdgfrb*	EVM0022774	Platelet-derived growth factor receptor beta	3.89
*Flt1*	EVM0007297	FMS-like tyrosine kinase 1	3.04
*Dusp*	EVM0020294	Dual specificity MAP kinase phosphatase	2.04
*Hspb1*	EVM0002084	Heat shock protein beta-1	4.37
*Kdr*	EVM0020836	Kinase insert domain protein receptor	2.96
*Ntrk2*	EVM0017245	Neurotrophic tyrosine kinase receptor type 2	4.74

## Data Availability

Not applicable.

## Supplementary Materials

The following are available online at https://www.mdpi.com/2076-2615/11/3/765/s1, Table S1: RNA purity of 27 samples of *S. argus*; Table S2: Quantitative real-time PCR (qRT-PCR) primer sequences data; Table S3: Top ten significant KEGG signaling pathways from three different groups.
