# Supplementary material for: Comparative Transcriptome Analysis Identifies Candidate Genes Related to Black-Spotted Pattern Formation in Spotted Scat (Scatophagus argus)
Source: Animals (Basel). 2021 Mar 10;11(3):765. doi: 10.3390/ani11030765 (PMC8001731; doi:10.3390/ani11030765)
Supplement: Supplementary file 1 [file animals-11-00765-s001.pdf]

Supplementary Table legends

Table S1 RNA purity of 27 samples of *S. argus*.

Serial number	sample name	OD260/280	OD260/230	RIN
1	black-spotted skin1	1.895	1.143	9.1
2	black-spotted skin2	1.542	0.685	8.3
3	black-spotted skin3	1.542	0.925	8
4	black-spotted skin4	1.667	0.494	7.5
5	black-spotted skin5	1.842	0.7	8.5
6	black-spotted skin6	1.423	0.649	8.3
7	black-spotted skin7	2.108	1	5.8
8	black-spotted skin8	2.394	2.135	6.5
9	black-spotted skin9	1.481	0.597	8.7
10	non-spotted skin1	2.269	0.573	7.6
11	non-spotted skin2	2.208	1.169	7.8
12	non-spotted skin3	2.318	1.594	7.9
13	non-spotted skin4	2.289	1.048	7.7
14	non-spotted skin5	1	0.196	8.6
15	non-spotted skin6	1.806	0.903	8.3
16	non-spotted skin7	2.171	0.685	6.6
17	non-spotted skin8	2.184	1.566	7.3
18	on-spotted skin9	1.756	1.091	8.2
19	caudal fin 1	1.581	0.916	7
20	caudal fin 2	1.924	1.322	6.9
21	acaudal fin 3	1.929	1.763	8.9
22	caudal fin 4	1.921	1.287	8.7
23	caudal fin 5	1.898	1.358	9.1
24	caudal fin 6	1.907	1.517	8.5
25	caudal fin 7	1.963	1.423	8.3
26	caudal fin 8	1.938	1.607	8.3
27	caudal fin 9	1.978	1.675	8.9

Table S2 Quantitative real time PCR (qRT-PCR) primer sequences data.

NO	Gene name	Primer name	Sequence (5′-3′)
1	*Sp6*	*Sp6-f*	AAGTTCAGCTGCGCTCTATGTCCT
		Sp6-r	CCATCTCCATTTACTCTCTCCTCC
2	*Slc6a6s*	*Slc6a6s-f*	AGACCAAGGTGGAAAAGAGGGAAC
		Slc6a6s-r	AAGAAACAGTAAGGCAACAGGAAG
3	*Efna*	*Efna-f*	AGGAGGATGGGAGTGGAGGAGAGG
		Efna-r	GTGTTATAGTGTGGGCAGTAGATG
4	*Actc1*	*Actc1-f*	GGGTGTCATGGTCGGTATGGGTCA
		Actc1-r	CAGCTCGTTGTAGAAGGTGTGGTG
5	*Tyrp1*	*Tyrp1-f*	AACAACACAGAGACCAGTCCCATC

		Tyrp1-r	GCTCCAAACAGTCCAACACATCCT
6	*Foxd*	*Foxd-f*	GACATGTTTGAGAACGGGAGTTTT
		Foxd-r	GCGTGGTGATGGAGGTAGGGATAA
7	*Mart-1*	*Mart-1-f*	GGGCATAGTTCTGCTGGTGGTCAT
		Mart-1-r	GCCATTTTGTTGTCTGCTGAAGGT
8	*Mapk4*	*Mapk4-f*	CAACCCCTCTCGTCTACTTCCTCC
		Mapk4-r	CGTCAACTTTCTCCTTATTCTGCC
9	*Hsp70*	*Hsp70-f*	ACACCAGCCAAGCCCTCACAGCAG
		Hsp70-r	CGTCCAAGCGAAGCGTCCCACACT
10	*Adcy5*	*Adcy5-f*	CTGTCTTCGTTCTCGCCCTCTACC
		Adcy5-r	CCTCCATCTCCTCTTTCTCTTCTG
11	*Gdf5*	*Gdf5-f*	AGTAGTGAAGATGCAGATGAGGCC
		Gdf5-r	CAGATGTGAACGGATGGGGAAGTC
12	*Mitf*	*Mitf-f*	GGAGCGGCGGGAGCAGCAGAGACA
		Mitf-r	CTGGGCGGCAGGTGGTAAAGTGGG
13	*Pmel*	*Pmel-f*	GCTGCTGGTTTTGGCTTTTACTTC
		Pmel-r	GTCAGGGTGGGTCCATCATTTTTC
14	*Pax3_7*	*Pax3_7-f*	ATGGATGAAGTGTCAGATGTGGAG
		Pax3_7-r	GGTATAGATGTCAGGGTAGTGGGT

Table S3 Top ten significant KEGG signaling pathway from different three groups. (1) Black spotted skin VS Non-spotted skin; (2) Black spotted skin VS Caudal fin; (3) Caudal fin VS Non-spotted skin.

Comparison	KEGG ID	Pathway description	counts	P value
Black spotted skin VS Non-spotted skin	dre00190	Oxidative phosphorylation	39	7.37E-21
	dre04260	Cardiac muscle contraction	28	3.31E-16
	dre04510	Focal adhesion	33	4.71E-08
	dre04512	ECM-receptor interaction	16	3.27E-06
	dre04261	Adrenergic signaling in cardiomyocytes	25	3.85E-06
	dre04020	Calcium signaling pathway	27	1.70E-05
	dre04371	Apelin signaling pathway	21	3.77E-05
	dre04270	Vascular smooth muscle contraction	18	0.000181
	dre04080	Neuroactive ligand-receptor interaction	29	0.000222
	dre00071	Fatty acid degradation	8	0.001637
Black spotted skin VS Caudal fin	dre00190	Oxidative phosphorylation	44	6.54E-19
	dre04260	Cardiac muscle contraction	33	1.63E-16
	dre04020	Calcium signaling pathway	42	7.44E-09
	dre04510	Focal adhesion	40	4.69E-07
	dre01200	Carbon metabolism	28	3.74E-06
	dre04261	Adrenergic signaling in cardiomyocytes	31	8.62E-06
	dre00010	Glycolysis / Gluconeogenesis	18	3.43E-05
	dre04512	ECM-receptor interaction	18	3.43E-05
	dre00071	Fatty acid degradation	11	0.000344
	dre04080	Neuroactive ligand-receptor interaction	38	0.00051
Caudal fin VS Non-spotted skin	dre00010	Glycolysis / Gluconeogenesis	10	9.81E-07
	dre00030	Pentose phosphate pathway	6	2.27E-05
	dre01230	Biosynthesis of amino acids	7	0.00054
	dre04020	Calcium signaling pathway	11	0.000562
	dre01200	Carbon metabolism	9	0.000648
	dre00051	Fructose and mannose metabolism	5	0.000879
	dre04260	Cardiac muscle contraction	6	0.001793
	dre04512	ECM-receptor interaction	6	0.00244
	dre00230	Purine metabolism	8	0.003074
	dre04510	Focal adhesion	10	0.004334
